# Undergraduate psychiatric education: current situation and way forward

**DOI:** 10.1192/bji.2021.48

**Published:** 2022-05

**Authors:** Gaia Sampogna, Hussien Elkholy, Franziska Baessler, Bulent Coskun, Mariana Pinto da Costa, Rodrigo Ramalho, Florian Riese, Andrea Fiorillo

**Affiliations:** 1 MD, PhD, Assistant Professor of Psychiatry, Department of Psychiatry, University of Campania ‘L. Vanvitelli’, Naples, Italy. Email gaia.sampogna@gmail.com; 2 MBBCh, MSc, MD, MRCPsych, Associate Professor and Consultant of Psychiatry, Neurology and Psychiatry Department, Faculty of Medicine, Ain Shams University, Cairo, Egypt; 3 Dr, MME, Department of General Internal and Psychosomatic Medicine, Center for Psychosocial Medicine, Heidelberg University Hospital, Germany; 4 Retired Faculty Member, Kocaeli University Medical School, Basiskele, Kocaeli, Turkey; 5 MD, MSc, PhD, Consultant Psychiatrist at South London and Maudsley NHS Foundation Trust and Senior Lecturer at the Institute of Psychiatry, Psychology & Neuroscience, King's College London, London, UK; 6 MD (Psychiatry), PhD, Department of Social and Community Health, School of Population Health, University of Auckland, New Zealand; 7 MD, Research Fellow, University Research Priority Program (URPP) Dynamics of Healthy Aging, University of Zurich, Switzerland; 8 MD, PhD, Full Professor of Psychiatry, Department of Psychiatry, University of Campania ‘L. Vanvitelli’, Naples, Italy

**Keywords:** Education and training, psychiatric education, recruitment, psychiatry programmes, mental health

## Abstract

Undergraduate psychiatric education is essential for the training of medical students and for their recruitment into psychiatry. A significant shortage of graduates choosing a career in psychiatry has been recently documented, and this trend might have many causes. When medical students have positive experiences of teaching, elective placements and exposure to psychiatric patients, their attitudes towards psychiatry are significantly better. Therefore, there is a need to improve the quality of undergraduate training courses in psychiatry. Innovative teaching strategies are suggested, including the use of movies, virtual reality, simulated patients and multiprofessional training wards.

Undergraduate education in mental health is an essential part of the training of medical students. Given the high prevalence of mental health problems in the general population, all practitioners should have the basic skills to identify and manage mental disorders.^1^ According to the World Psychiatric Association (WPA), undergraduate psychiatric education aims to improve the quality of mental health education and, consequently, the quality of care provided to people with mental disorders.

Furthermore, high-quality undergraduate education in psychiatry is crucial for the future recruitment of medical students into psychiatry as a specialty. In the past 10–15 years the percentage of graduates choosing a career in psychiatry has been significantly reduced.^2^ Possible explanations for this negative trend include the low quality of teaching, stigma towards people with mental disorders and negative attitudes of medical doctors towards psychiatry.^3^ In particular, it could be that some aspects of the psychiatric discipline, such as the use of compulsory treatment and the presence of negative stereotypes attached to psychiatrists, may have an adverse effect on both recruitment and retention of medical students in psychiatry.^3–5^ In some European countries, students may consider a placement in psychiatry as ‘low priority’.^6^

However, students’ attitudes towards the discipline change if they have positive teaching experiences, elective placements and direct contact with patients with mental disorders. Several strategies have been proposed for improving the attitudes of medical students towards psychiatry. These include direct contact with patients (particularly those who get better after treatment), an emphasis on the evidence-based treatments and approaches used in psychiatry, and a close relationship with consultants and senior trainees during their education. Therefore, to overcome stigma and the negative image of the discipline, in 2009, the Royal College of Psychiatrists in the UK suggested organising periodic meetings between medical students and licensed psychiatrists throughout medical school and the development of an *ad hoc* mentoring programme.^7^

Furthermore, receiving an adequate undergraduate psychiatric education may be useful not only for students interested in psychiatry, but it could also help those who want to pursue a different medical career. In particular, undergraduate psychiatric education may teach them the communication skills, and explain concepts and thought processes necessary to understand patients and their disorders from biological, psychological and sociocultural viewpoints.

Improving the quality of undergraduate education can support the process of de-stigmatisation of psychiatry, since medical students can develop correct and unbiased opinions regarding the discipline, which in turn can play a significant role in ameliorating the healthcare services provided to patients with mental health problems (or related physical complaints) in other medical settings.

Taking a worldwide perspective, this paper aims to: (a) describe the characteristics of undergraduate training in psychiatry; (b) discuss the most relevant unmet needs of undergraduate training in psychiatry in medical schools; (c) outline some innovative strategies to improve undergraduate psychiatric education.

## Characteristics of undergraduate training in psychiatry

Undergraduate psychiatric training greatly varies across different countries and university sites. Some medical schools include psychiatry programmes as optional, and others have only 2 weeks of mandatory formal training in psychiatry.^8^ A recent survey promoted by the International Federation of Medical Students’ Associations (IFMSA) in collaboration with the WPA noted that psychiatry education is mandatory in 81 out of the 83 surveyed countries, and is an elective course only in two countries (Ethiopia and Nigeria).^9^ Furthermore, the duration of classes on theory significantly varies across countries (e.g. from just 1 day to more than 30 days); Nigeria and Burkina Faso do not have any practical classes at all. Even in Western countries, some medical schools offer only 4 weeks of training in psychiatry.^9^ Undergraduate psychiatric training in the USA is longer than it is in Asian and Pacific regions. Methods for evaluating students’ knowledge and competencies also vary among countries, although the most common way of assessing it is multiple choice questions. The format of teaching does not significantly vary, with most medical schools offering a combination of lectures, bedside teaching and computer-based learning.^8^ Overall, the survey conducted by IFMSA shows that undergraduate psychiatric education is not given enough prominence in undergraduate medical curricula.

## Unmet needs and the way forward for undergraduate training in psychiatry

A number of unmet needs in undergraduate psychiatric education have recently been highlighted in the literature. In particular, the limited availability of tutors, highly trained faculty members and clinicians at several sites is adversely affecting the quality of provided and perceived undergraduate training.^10^

Second, in some contexts, limited resources are a significant obstacle to good-quality training, especially in relation to access to adequate facilities and evidence-based educational materials.

Many traditional educational methods are not easily applied in undergraduate psychiatric education (e.g. some patients may be reluctant to disclose their mental problems to students because of delusional thoughts; others may feel that the presence of a third person breaks the therapeutic alliance with their psychotherapist). Furthermore, in many sites, students have limited access to psychiatric patients at the bedside, and thus gain little practical knowledge of many psychiatric disorders.^11^

Therefore, novel methods to teach psychiatry should be put in place and trialled. It is beyond the scope of this paper to systematically review all the innovative teaching models used in psychiatry, so we have selected some of the most promising approaches to outline here.

Simulation techniques offer one innovative solution. Techniques such as student role-play and simulated patients have been found to improve the communication skills and empathy of undergraduate medical students and seem to be very well accepted.^12^ These techniques are used to teach history taking and formulating a management plan and can depict challenging clinical situations (e.g. acutely agitated patients) or rare syndromes, which would otherwise be neglected during training.^13^

Another innovative solution is the use of movies as an educational tool. Watching movies and videos with students may allow them to discuss misconceptions about mental disorders in a relaxed setting (i.e. not in the patient's presence) and whether the clinical descriptions are accurate or not. Using videos to teach particular topics to medical students has been shown to result in improved recall of those situations.^14^

The Psychiatry Early Experience Programme (PEEP) was proposed in 2015 in the UK. Medical students are paired with specialists in psychiatry, to shadow them while on-call and for one regular day shift during each of their jobs.^15^ By joining the programme, students are exposed to a wide range of mental health problems and develop insight into psychiatric trainees’ work. The students can also attend lectures given by psychiatric experts and clinical sessions with patients. The programme looks very promising, as confirmed by the fact that many students who joined it developed more positive attitudes towards psychiatry and seemed less reluctant to choose psychiatry as a specialty.^15^

In 2019, the American Psychiatric Association launched the Psychiatry Student Interest Group Network (PsychSIGN), a networking initiative that includes students interested in psychiatry. It provides resources and mentoring opportunities for students to help them to deepen their interest in the field.^9,^^16^

Interprofessional training wards offer another innovative teaching opportunity. These enable students and trainees from different health professions to work in collaboration to manage the medical treatment and rehabilitation of real-life patients, taking a multidisciplinary perspective.

Finally, another aspect to be improved is related to providing feedback to medical students at the end of their rotation in psychiatry. A recent study carried out in Sweden using a structured feedback tool found that medical students were more satisfied at the end of the rotation period if they had received structured feedback on their internship.^17^

## Conclusions

Undergraduate training in psychiatry is essential for psychiatric education and practice. Providing good-quality undergraduate training increases students’ interest in mental health, reduces stigma towards people with mental illness and increases students’ confidence in working with people with mental health problems.^1–3,18^

Initiatives aiming at improving psychiatric education during the early years of medical school have been well received and have been found to be effective in changing the attitudes of medical students towards the discipline. Novel techniques using virtual reality, movies and simulation can help increasing the psychiatric knowledge and practical skills of undergraduate medical students, without affecting patient confidentiality and the therapeutic alliance.

## Data Availability

Data availability is not applicable to this article as no new data were created or analysed in this study.
