# Undergraduate psychiatric education: current situation and way forward – CORRIGENDUM

**DOI:** 10.1192/bji.2021.61

**Published:** 2022-05

**Authors:** Gaia Sampogna, Hussien Elkholy, Franziska Baessler, Bulent Coskun, Mariana Pinto da Costa, Rodrigo Ramalho, Florian Riese, Andrea Fiorillo

**Keywords:** Education and train-ing, psychiatric education, recruitment, psychiatry pro-grammes, mental health

The authors would like to make two corrections to the above paper.

1. The correct affiliation details for Mariana Pinto Da Costa are as follows: Mariana Pinto Da Costa, MD, MSc, PhD, Consultant Psychiatrist at South London and Maudsley NHS Foundation Trust and Senior Lecturer at the Institute of Psychiatry, Psychology & Neuroscience, King's College London, London, UK
2. The correct wording in the declaration of interests should be “MPC is a member of the Editorial Board of The British Journal of Psychiatry and of BJPsych Advances” and “HE is a member of the Editorial Board of the British Journal of Psychiatry International”.

The authors apologise for these errors.
